# Equine Foods

**Published:** 1895-01

**Authors:** 


### EQUINE FOODS.

THE earliest mention of oats in China was A. D. 618.

Oats were not known to the Hebrews or the Egyptians.

Millet is pre-historic in South Europe, Egypt, and Asia.

"Spurred rye" is one of the most deadly poisons known.

Maize has been found in the most ancient Peruvian tombs.

The straw of rye is often of far more value than the grain.

Maize has probably more enemies than any other species of grain.

Some valuable use has been found for every part of the maize plant.

It is said that mules fed on corn that has the smut will lose their hoofs.

Millet is sowed by the Chinese Emperor in a solemn ceremonial every year.

The Greeks had oats 200 B. C., but used them only as food for their horses.

Japan has developed a variety of maize with leaves striped with white.

